# The Sacrifice of the Civilian

**Published:** 1918-09-14

**Authors:** 


### THE SACRIFICE OF THE CIVILIAN.

If a nation at war were like a man in a street row, and needed to devote mind and resources wholly to the fight, and neglect for a few short moments all other interests, then we could understand more easily an official policy lately animadverted upon by two important civilian bodies whose patriotism is not open to question. Recently the Leeds Chamber of Commerce again complained of the action of the Priority Department of the War Munitions Board in refusing a supply of thermometers for Leeds Hospital, and also the sanction to install a fan in a Harrogate operating theatre. The department's reply to the first complaint had been merely to take exception to any adverse criticism of their action as being unpatriotic, and out of place at present. Again, the Yorkshire West Riding National Insurance Committee, whose administrative area is one of the largest, most populous, and most important in the kingdom, about the same time agreed to a resolution of their Medical Service and Sanatorium Benefits Sub-Committee which took the following form:—

> That this committee place on record their strong opinion that the time has now arrived when the Government should make provision for the treatment of tuberculous soldiers either by releasing building materials and labour, so that suitable accommodation can be erected, or by accepting direct responsibility for treatment, thus relieving for the use of the civilian population the institutions at present so largely occupied by tuberculous soldiers to the exclusion of the civilian population.

Now in both these instances the official view seems to be that a great four years' war, the biggest in history, is comparable with a five minutes' bout of fisticuffs; that the immediate offensive is everything, and conservation of resources nothing—in short, that national health and *morale* are inconsiderable. Even for officialism, this view is surprisingly short-sighted. Take only the case of the recruit who needs some surgical operation to move him up a grade. Cannot really a couple of joiners be spared to put in a ventilating fan where he is to be operated upon? Must overworked house surgeons and nurses be delayed while two or three clinical thermometers are going the round of a whole ward? Consider again the great increase of tuberculosis amongst munition girls. Just now a tuberculous civilian may easily have to wait three or more months for admission to a sanatorium, after getting upon the list. Any readmission of a civilian is practically unknown, while few get the chance of an extended stay. Of course everyone is agreed that tuberculous combatants should have priority; and at present, in the area named, they are admitted within about a fortnight of examination and selection, the said selection being much less stringent than that of non-combatants. Further, they may and do stay in an institution as much as two years. Now, with the current available accommodation, this liberality, rather this justice, to the combatant means injustice to the civilian, to the ordinary National Insurance contributor who falls phthisical, perhaps in war employment, and naturally looks for help in return for years of regular payments. That injustice, too, may easily, of course, take the outrageous form of a death sentence. It is a very plain and pregnant issue, this which the Insurance Committee named has just raised. And, since the warrior cannot be put off with anything less than what he is getting, or the bacillus of Koch be persuaded to forgo the chance of havoc which war-time privations create for it, the only solution of the problem seems to lie in the direction indicated in the very unambiguous resolution we have quoted. More provision must be made against the home foe tuberculosis, whatever happens to uncompleted shipways or aerodromes, for weakness at home is the worst kind of weakness.

Is not this scientific conclusion for once surprisingly like the humanist's view? For does not, or rather—for nowadays he is rare—did not that cultured individual warn us that all great empires have fallen, not from injury to their outlying provinces, but from a blow at their heart? And if health and good temper at home suffer, it means a very heavy blow indeed. This is one of the things that go to prove the necessity of a Ministry of Health headed by a man of high standing, not at the beck and call of any newspaper, and not concerned to become a popular figure. Such an one it should not take long to get the jilted buildings we have all read of put to useful purpose after all, the while existing tuberculosis institutions in the great industrial districts were promptly enlarged. As for the staffing, he would also have something to say regarding the present allocation, military or civilian, of medical men and of nurses.

